# Leukemic transformation among 1306 patients with primary myelofibrosis: risk factors and development of a predictive model

**DOI:** 10.1038/s41408-019-0175-y

**Published:** 2019-01-25

**Authors:** Rangit R. Vallapureddy, Mythri Mudireddy, Domenico Penna, Terra L. Lasho, Christy M. Finke, Curtis A. Hanson, Rhett P. Ketterling, Kebede H. Begna, Naseema Gangat, Animesh Pardanani, Ayalew Tefferi

**Affiliations:** 1 Departments of Divisions of Hematology, Mayo Clinic, Rochester, MN USA; 2 Departments of Hematopathology, Mayo Clinic, Rochester, MN USA; 3 Laboratory Genetics and Genomics, Departments of Internal Medicine and Laboratory Medicine, Mayo Clinic, Rochester, MN USA

## Abstract

Among 1306 patients with primary myelofibrosis (PMF), we sought to identify risk factors that predicted leukemic transformation (LT) in the first 5 years of disease and also over the course of the disease. 149 (11%) LT were documented; patients who subsequently developed LT (*n* = 149), compared to those who remained in chronic phase disease (*n* = 1,157), were more likely to be males (*p* = 0.02) and display higher circulating blasts (*p* = 0.03), *ASXL1* (*p* = 0.01), *SRSF2* (*p* = 0.001) and *IDH1* (*p* = 0.02) mutations. Logistic regression analysis identified *IDH1*, *ASXL1* and *SRSF2* mutations, very high-risk karyotype, age > 70 years, male sex, circulating blasts ≥ 3%, presence of moderate or severe anemia and constitutional symptoms, as predictors of LT in the first 5 years of diagnosis. Time-to-event Cox analysis confirmed LT prediction for *IDH1* mutation (HR 4.3), circulating blasts ≥ 3% (HR 3.3), *SRSF2* mutation (HR 3.0), age > 70 years (HR 2.1), *ASXL1* mutation (HR 2.0) and presence of moderate or severe anemia (HR 1.9). HR-based risk point allocation resulted in a three-tiered LT risk model: high-risk (LT incidence 57%; HR 39.3, 95% CI 10.8–114), intermediate-risk (LT incidence 17%; HR 4.1, 95% CI 2.4–7.3) and low-risk (LT incidence 8%). The current study provides a highly discriminating LT predictive model for PMF.

## Introduction

Primary myelofibrosis (PMF) is an aggressive myeloid malignancy currently listed under the World Health Organization (WHO) category of myeloproliferative neoplasms (MPN)^1^. PMF represents a stem cell-derived clonal expansion of myeloid cells that often harbor one of three driver mutations, including *JAK2*, *CALR* and *MPL*. PMF is morphologically characterized by abnormal megakaryocyte proliferation that is often accompanied by reticulin fibrosis. Patients with PMF typically display severe anemia, marked hepatosplenomegaly and profound constitutional symptoms. Other complications of the disease include cachexia, thrombosis, bleeding and leukemic transformation (LT). Overall survival (OS) in PMF is estimated at 6 years and can range between a few months to over 20 years, depending on the presence or absence of specific clinical and genetic risk factors^2–4^. Current treatment in PMF includes allogeneic stem cell transplant (allo-SCT), which is the only treatment modality with the potential to cure the disease or prolong survival^5^. Other treatment approaches in PMF are mostly palliative and include drug therapy (e.g. JAK2 inhibitors), splenectomy and involved field radiation therapy^6^.

Taking the above into consideration, the primary objective in developing a treatment strategy for the individual patient with PMF is to establish the timing of allo-SCT. The particular task is often accomplished by considering risk level, according to previously established risk models for OS. At present, these include the genetically-inspired prognostic scoring system (GIPSS)^3^ and the mutation- and karyotype-enhanced prognostic scoring system (MIPSS70 + version 2.0)^4^. GIPSS relies on genetic risk factors only, including karyotype, driver mutations and other mutations, including *ASXL1*, *SRSF2* and *U2AF1* Q157. MIPSS70 + version 2.0 utilizes the same genetic risk factors used in GIPSS but also incorporates three specific clinical risk factors, including constitutional symptoms, presence of severe/moderate anemia and ≥ 2% circulating blasts. The main objective for the current study was to develop a robust LT predictive model that complements GIPSS and MIPSS70 + version 2.0 and thus further facilitates treatment decision-making in PMF; in this regard, it is to be recalled that, in the context of GIPSS/MIPSS70 + , leukemia-free survival (LFS) was previously shown to be affected by karyotype, *SRSF2* and *ASXL1* mutations, platelet count < 100 × 10^9^/l and circulating blasts ≥ 2%^3,7^.

## Methods

The current study was approved by the institutional review board of the Mayo Clinic, Rochester, MN, USA. The study population consisted of consecutive patients with PMF seen at our institution between April 26, 1976 and November 21, 2017. Diagnoses of PMF and LT were confirmed by both clinical and bone marrow examinations, in line with the 2016 WHO criteria; specifically, LT required presence of ≥ 20% blasts in the peripheral blood (PB) or bone marrow (BM)^1^. Data was collected retrospectively corresponding to the time of first referral which in the majority of cases was at the time of or within the first year of diagnosis. All patients were followed until death or last follow-up as assessed by medical records or through direct contact with patients or their physicians. Data collection was updated as of April 2018. The determination of prognostically relevant mutations was made by next generation sequencing (NGS)-derived mutation information^8,9^. Cytogenetics data were analyzed using standard techniques and reported in conformity with the International System for Human Cytogenetic Nomenclature criteria^10^.

Variables evaluated included those that are currently listed in MIPSS70^7^, MIPSS70 + version 2.0^4^ and GIPSS^3^, as well as age ( ≤ 70 vs > 70 years) and sex. Constitutional symptoms were defined as:^1^ weight loss > 10% of baseline during the year before the diagnosis, or^2^ unexplained excessive sweats, or^3^ fever persisting for at least a month^11^. Karyotype was designated as favorable, unfavorable or very high-risk (VHR), according to the recently published revised three-tiered cytogenetics risk model;^12^ VHR karyotype was defined as chromosomal abnormalities with single/multiple abnormalities of −7, i(17q), inv(3)/3q21, 12p−/12p11.2, 11q−/11q23, or other autosomal trisomies not including + 8/ + 9 (e.g., + 21, + 19)^12^. Sex-adjusted values for hemoglobin were categorized as severe anemia, defined by hemoglobin levels of < 8 g/dl in women and < 9 g/dl in men, and moderate anemia, defined by hemoglobin levels of 8–9.9 g/dl in women and 9–10.9 g/dl in men^13^. High molecular risk (HMR) mutations included *ASXL1*, *SRSF2*, *U2AF1* Q157, *IDH1/2* and *EZH2*^14,15^.

Statistical analyses considered clinical and laboratory data collected at the time of initial PMF diagnosis or Mayo Clinic referral point. Continuous variables are presented as median (range) and categorical variables as frequency (percentage). The differences in the distribution of continuous variables between categories were compared using the Mann–Whitney or Kruskal–Wallis test. Categorical variables were compared using the *χ*^2^ test. Logistic regression statistics was employed in order to identify predictors of LT at 5 years (i.e., early events) from initial diagnosis/referral; in the particular method, patients with documented LT within 5 years were “uncensored” while those followed up for at least 5 years, without developing LT, were “censored”; the analysis excluded patients without LT and not followed for at least 5 years. In addition, Cox regression analysis was performed to identify risk factors for overall leukemia-free survival (LFS). The Kaplan–Meier method was used to construct time-to-leukemia curves, which were compared by the log-rank test. *P* values of < 0.05 were considered significant. In order to develop LT predictive model, HR-based risk point allocation was employed and predictive accuracy was compared to those of GIPSS and MIPSS70 + version 2.0, using Akaike Information Criterion (AIC) and receiver operating characteristic (ROC) curve-derived area under the curve (AUC) estimates. The JMP® Pro 13.0.0 software from SAS Institute, Cary, NC, USA, was used for all calculations.

## Results

The current study included 1306 consecutive patients with PMF (median age 65 years, range 19-92; 63% males) seen at the Mayo Clinic between April 26, 1976 and November 21, 2017. Details of presenting clinical and laboratory features are outlined in Table 1. Among evaluable patients, sex-adjusted moderate or severe anemia was present in 54% of the patients at time of PMF diagnosis, thrombocytopenia < 100 × 10^9^/l in 23%, leukocytosis > 25 × 10^9^/l in 15%, circulating blasts ≥ 3% in 17%, constitutional symptoms in 29%, thrombosis history in 16%, VHR karyotype in 6% and other unfavorable karyotype in 17%. Driver mutation distribution was 67% *JAK2*, 16% *CALR* type 1/like, 3% *CALR* type 2/like, 6% *MPL* and 8% triple-negative (Table 1). Also in evaluable patients, mutational frequencies were 41% for *ASXL1*, 14% *SRSF2*, 2% *IDH1*, 4% *IDH2*, 4% *EZH2*, 15% *U2AF1* and 10% *U2AF1* Q157. DIPSS risk distribution was evaluable in 1265 patients and included 9% high risk, 39% intermediate-2 risk, 37% intermediate-1 risk and 15% low risk (Table 1). GIPSS risk distribution was evaluable in 560 patients and showed 25% high risk, 30% intermediate-2 risk, 35% intermediate-1 risk and 9% low risk (Table 1). MIPSS70 + version 2.0 was evaluable in 513 patients and showed 20% very high risk, 41% high risk, 19% intermediate risk, 16% low risk and 4% very low risk.

**Table 1:** Clinical and laboratory characteristics, at time of initial diagnosis of primary myelofibrosis, of 1306 patients, stratified by whether or not they developed leukemic transformation during their clinical course

Variables	All patients (*n* = 1306)	Patients who transformed into acute myeloid leukemia during their clinical course (*n* = 149)	Patients who remained in chronic phase disease at last follow-up (*n* = 1157)	*P* value
Age in years; median (range)	65 (19–92)	64 (32–85)	65 (19–92)	0.2
Age > 70 years; *n* (%)	382 (29)	35 (23)	347 (30)	0.1
Males; *n* (%)	820 (63)	106 (71)	714 (62)	**0.02**
Hemoglobin, g/dl; median (range) “*N*” evaluable = 1298	10.2 (3.8–17.5)	10.2 (6.1–15.2)	10.3 (3.8–17.5)	0.7
Hemoglobin < 10 g/dl; *n* (%) “*N*” evaluable = 1298	608 (47)	69 (48)	539 (47)	0.8
Sex and severity adjusted anemia categories				0.6
“*N*” evaluable = 1298				
Mild/no anemia; *n* (%)	591 (46)	63 (44)	528 (46)	
Moderate/severe anemia; *n* (%)	707 (54)	81 (56)	626 (54)	
Transfusion dependent; *n* (%) “*N*” evaluable = 1299	417 (32)	38 (26)	379 (33)	0.1
Platelets, ×10^9^/l; median (range) “*N*” evaluable = 1299	225 (6–2400)	202 (10–2399)	230 (6–2400)	0.09
Platelets < 100 × 10^9^/l; *n* (%) “*N*” evaluable = 1299	294 (23)	38 (26)	256 (22)	0.2
Leukocytes, ×10^9^/l; median (range) “*N*” evaluable = 1298	8.8 (0.8–249)	10 (1.1–249)	8.8 (0.8–236)	0.5
Leukocytes > 25 × 10^9^/l; *n* (%) “*N*” evaluable = 1298	189 (15)	23 (16)	166 (14)	0.6
Circulating blasts %; median (range) “*N*” evaluable = 1283	0 (0–18)	1 (0–18)	0 (0–18)	**0.03**
Circulating blasts ≥ 3%; *n* (%) “*N*” evaluable = 1283	217 (17)	34 (24)	183 (16)	**0.02**
Palpable splenomegaly; *n* (%) “*N*” evaluable = 1260	902 (72)	94 (70)	808 (72)	0.6
Bone marrow fibrosis grade (2 or above); *n* (%) “*N*” evaluable = 793	646 (81)	82 (79)	564 (82)	0.4
Constitutional symptoms; *n* (%) “*N*” evaluable = 1302	375 (29)	45 (31)	330 (29)	0.5
History of any thrombosis at or prior to diagnosis; *n* (%) “*N*” evaluable = 1299	208 (16)	15 (11)	193 (17)	0.05
History of venous thrombosis at or prior to diagnosis; *n* (%) “*N*” evaluable = 1298	92 (7)	5 (4)	87 (8)	0.08
History of arterial thrombosis at or prior to diagnosis; *n* (%) “*N*” evaluable = 1299	136 (10)	12 (8)	124 (11)	0.4
Karyotype				0.2
“*N*” evaluable = 1218				
Favorable; *n* (%)	931 (76)	91 (71)	840 (77)	
Unfavorable; *n* (%)	212 (17)	26 (20)	186 (17)	
VHR; *n* (%)	75 (6)	12 (9)	63 (6)	
DIPSS risk stratification				**0.005**
“*N*” evaluable = 1265				
High risk; *n* (%)	111 (9)	7 (6)	104 (9)	
Intermediate risk-2; *n* (%)	501 (39)	50 (42)	451 (39)	
Intermediate risk-1; *n* (%)	466 (37)	54 (46)	412 (36)	
Low risk; *n* (%)	187 (15)	7 (6)	180 (16)	
GIPSS risk stratification				0.07
“*N*” evaluable = 560				
High risk; *n* (%)	142 (25)	21 (29)	121 (25)	
Intermediate risk-2; *n* (%)	169 (30)	29 (40)	140 (29)	
Intermediate risk-1; *n* (%)	198 (35)	18 (25)	180 (37)	
Low risk; *n* (%)	51 (9)	4 (6)	47 (10)	
MIPSS70 + version 2.0 risk stratification				**0.02**
“*N*” evaluable = 513				
Very high risk; *n* (%)	104 (20)	15 (17)	89 (21)	
High risk; *n* (%)	209 (41)	49 (56)	160 (38)	
Intermediate risk; *n* (%)	97 (19)	9 (10)	88 (21)	
Low risk; *n* (%)	80 (16)	13 (15)	67 (16)	
Very low risk; *n* (%)	23 (4)	2 (2)	21 (5)	
Driver mutational status				0.06
“*N*” evaluable = 897				
*JAK2*; *n* (%)	603 (67)	48 (54)	555 (69)	
*CALR type 1/like*; *n* (%)	149 (16)	18 (20)	121 (15)	
*CALR type 2/like*; *n* (%)	31 (3)	4 (4)	27 (3)	
*MPL; n* (%)	54 (6)	7 (8)	47 (6)	
Triple-negative*; n* (%)	70 (8)	12 (13)	58 (7)	
*ASXL1* mutated; *n* (%) “*N*” evaluable = 596	246 (41)	41 (55)	205 (39)	**0.01**
*SRSF2* mutated; *n* (%) “*N*” evaluable = 597	83 (14)	21 (27)	62 (12)	**0.001**
*IDH1* mutated; *n* (%) “*N*” evaluable = 479	9 (2)	4 (6)	5 (1)	**0.02**
*IDH2* mutated; *n* (%) “*N*” evaluable = 479	18 (4)	5 (8)	13 (3)	0.07
*EZH2* mutated; *n* (%) “*N*” evaluable = 452	17 (4)	2 (3)	15 (4)	0.9
*U2AF1* mutated; *n* (%) “*N*” evaluable = 579	88 (15)	11 (15)	77 (15)	0.9
*U2AF1* Q157 mutated; *n* (%) “*N*” evaluable = 579	57 (10)	6 (8)	51 (10)	0.8
Allogeneic stem cell transplant; *n* (%)	68 (6)	4 (3)	64 (6)	0.2
Follow-up in years; median (range)	3.2 (0–31)	3.1 (0.3–20.2)	3.2 (0–31)	0.9
Deaths; *n* (%)	922 (71)	142 (95)	780 (67)	**<0.0001**

*DIPSS* dynamic international prognostic scoring system, *GIPSS* genetically-inspired prognostic scoring system, *MIPSS70* + *Version 2.0* mutation-enhanced international prognostic scoring system, *VHR* very high-risk karyotype

Bold values indicates significance indicator

Median follow-up was 3.2 years (range 0-31); during this time, a total of 149 (11%) cases of LT were documented. Comparison of clinical and laboratory features, recorded at the time of initial PMF diagnosis, between the patients who subsequently developed LT (*n* = 149) and those who remained in chronic phase disease at last follow-up (*n* = 1,157) reveled the former to be more likely to be males (*p* = 0.02) and display higher incidence of excess circulating blasts (*p* = 0.03), *ASXL1* (*p* = 0.01), *SRSF2* (*p* = 0.001) and *IDH1* (*p* = 0.02) mutations (Table 1).

We employed two separate statistical methods in order to assess the risk of developing LT (Table 2). The first method involved binary outcome analysis using logistic regression, in order to calculate the odds of developing LT in the first 5 years of disease (elaborated further in the *Methods* section). In univariate analysis, the logistic 5-year risk of LT was predicted by age > 70 years, male sex, moderate or severe anemia, thrombocytopenia < 100 × 10^9^/l, leukocytosis of > 25 × 10^9^/l, circulating blasts ≥ 3%, constitutional symptoms, VHR karyotype, absence of *CALR* type 1/like and mutations affecting *ASXL1*, *SRSF2*, *IDH1* and *IDH2* (odds ratio (OR) and 95% CI are provided in Table 2); multivariable logistic regression confirmed the independent prognostic contribution of *IDH1* mutation (OR 78.4), VHR karyotype (OR 57.6), *ASXL1* mutation (OR 15.1), age > 70 years (OR 13.3), *SRSF2* mutation (OR 8.5), male sex (OR 6.9), circulating blasts ≥ 3% (OR 5.4), presence of sex-adjusted moderate or severe anemia (OR 3.6) and constitutional symptoms (OR 3.1). A parallel time-to-event Cox analysis confirmed inferior LFS in patients with *IDH1* mutation (HR 4.3), *SRSF2* mutation (HR 3.0), *ASXL1* mutation (HR 2.0), circulating blasts ≥ 3% (HR 3.3), age > 70 years (HR 2.1), and presence of sex-adjusted moderate or severe anemia (HR 1.9).

**Table 2:** Univariate and multivariable analysis of clinical and genetic predictors of leukemic transformation in 1306 patients with primary myelofibrosis

	Predictors of leukemic transformation in the first 5 years of diagnosis (Logistic regression analysis)		Risk factors for leukemia-free survival (Cox analysis)	
Variables	Univariate analysis *P* value (OR, 95% CI)	Multivariable analysis *P* value (OR, 95% CI)	Univariate analysis *P* value (HR, 95% CI)	Multivariable analysis *P* value (HR, 95% CI)
Age in years	**<0.001**		**0.01** (1.02, 1–1.03)	
Age > 70 years	**0.003** (2.1, 1.3–3.3)	**<0.001** (13.3, 3.5–51.2)	0.4 (1.2, 0.8–1.7)	**0.03** (2.1, 1.1–3.8)
Gender (Male)	**<0.001** (2.8, 1.7–4.6)	**0.01** (6.9, 1.6–30.2)	**0.002** (1.7, 1.2–2.5)	
Sex and severity adjusted anemia				
“*N*” evaluable = 1298				
Moderate/Severe anemia	< **0.001** (3.1, 2.0–4.9)	**0.02** (3.6, 1.2–10.7)	< **0.001** (1.8, 1.3–2.6)	**0.02** (1.9, 1.1–3.3)
No/Mild anemia	Reference		Reference	
Platelets, ×10^9^/l “*N*” evaluable = 1299	**<0.001**		0.05 (0.2, 0.04–1.03)	
Platelets**<**100 × 10^9^/l “*N*” evaluable = 1299	**<0.001** (3.6, 2.1–6.1)		**0.001** (1.9, 1.3–2.8)	
Leukocytes, ×10^9^/l “*N*” evaluable = 1298	**<0.001**		**<0.001** (17, 3.9–51.4)	
Leukocytes > 25 × 10^9^/l “*N*” evaluable = 1298	**0.002** (3.4, 1.8–6.3)		**0.01** (1.8, 1.1–2.8)	
Circulating blasts % “*N*” evaluable = 1283	**<0.001**		**<0.001** (18.5, 7.3–41.6)	
Circulating blasts ≥ 3% “*N*” evaluable = 1283	**<0.001** (3.6, 2.2–6.1)	**0.009** (5.5, 1.5–19.6)	**<0.001** (2.6, 1.7–3.7)	**0.001** (3.3, 1.6–6.2)
Palpable splenomegaly “*N*” evaluable = 1260	0.4 (0.8, 0.5–1.3)		0.3 (0.8, 0.6–1.2)	
Bone marrow fibrosis grade (2 or above) “*N*” evaluable = 793	0.5 (0.8, 0.4–1.5)		0.6 (0.9, 0.6–1.5)	
Constitutional symptoms “*N*” evaluable = 1302	**<0.001** (2,5, 1.5–3.9)	**0.04** (3.1, 1.0–9.2)	**0.009** (1.6, 1.1–2.3)	
Any thrombosis at or prior to diagnosis “*N*” evaluable = 1299	0.3 (0.7, 0.3–1.4)		0.08 (0.6, 0.4–1.1)	
Venous thrombosis at or prior to diagnosis “*N*” evaluable = 1298	0.2 (0.5, 0.1–1.4)		**0.04** (0.4, 0.2–1)	
Arterial thrombosis at or prior to diagnosis “*N*” evaluable = 1299	0.8 (1.1, 0.5–2.2)		0.7 (0.9, 0.5–1.6)	
Presence of very high-risk (VHR) karyotype	**<0.001** (10.9, 3.7–32.3)	**0.005** (57.6, 3.3–994)	**<0.001** (3.6, 1.9–6.3)	
Karyotype				
“*N*” evaluable = 1218				
VHR	**<0.001** (12, 4–35.7)		**<0.001** (3.9, 2–7)	
Unfavorable	**0.08** (1.6, 0.9–2.9)		0.05 (1.6, 1–2.9)	
Favorable	Reference		Reference	
Driver mutational status				
“*N*” evaluable = 897				
*JAK2*	0.7 (0.9, 0.5–1.5)		0.2 (0.7, 0.5–1.1)	
*CALR*	0.2 (0.6, 0.3–1.3)		0.9 (0.9, 0.6–1.6)	
*MPL*	0.5 (1.3, 0.4–3.7)		0.5 (1.3, 0.5–2.6)	
Triple-negative	0.05 (2.2, 0.9–4.7)		0.06 (1.9, 0.9–3.3)	
*CALR type 1/like* absent	**0.045** (2.2, 1.0–4.9)		0.7 (1.1, 0.7–1.8)	
**Mutations**				
*ASXL1* mutated “*N*” evaluable = 596	**<0.001** (3.8, 2–7.2)	**<0.001** (15.1, 4.0–56.7)	**<0.001** (2.2, 1.4–3.5)	**0.01** (2.0, 1.2–3.3)
*SRSF2* mutated “*N*” evaluable = 597	**<0.001** (8.04, 3.7–17.2)	**0.002** (8.5, 2.2–32.2)	**<0.001 (3.5, 2.1–5.7)**	**0.001** (3.0, 1.6–5.3)
*IDH1* mutated “*N*” evaluable = 479	**0.005** (23.5, 2.5–217)	**0.002** (78.4, 5.3–1153)	**0.006** (6.2, 1.9–15.4)	**0.03** (4.3, 1.2–11.7)
*IDH2* mutated “*N*” evaluable = 479	**0.002** (9.9, 2.3–43.7)		**0.01** (3.1, 1.1–7.1)	
*EZH2* mutated “*N*” evaluable = 452	0.4 (1.9, 0.4–9.7)		0.7 (1.3, 0.2–4.2)	
*U2AF1* mutated “*N*” evaluable = 579	0.09 (2, 0.9–4.6)		0.3 (1.4, 0.7–2.7)	
*U2AF1* Q157 mutated “*N*” evaluable = 579	0.3 (1.8, 0.6–5.9)		0.5 (1.4, 0.5–3)	

**Abbreviation**: *OR* odds ratio, *HR* hazard ratio, *CI* confidence interval

Bold values indicates significance indicator

**Fig. 1:**
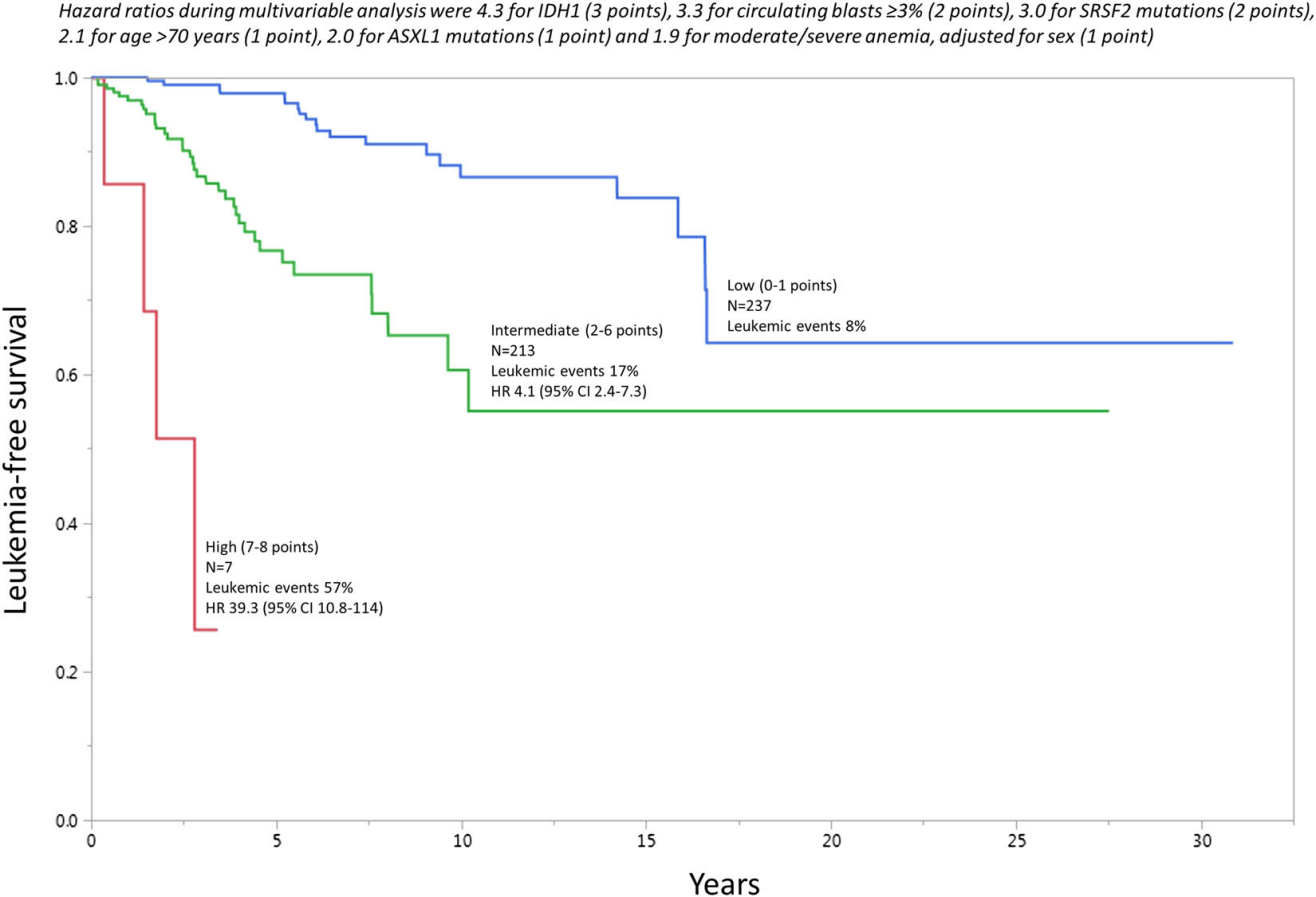
Leukemia-free survival among 456 patients with primary myelofibrosis who were informative for independent predictors of leukemic transformation (i.e., *IDH1*, *SRSF2* and *ASXL1* mutations; age > 70 years; circulating blasts ≥ 3%; moderate/severe anemia)

**Fig. 2:**
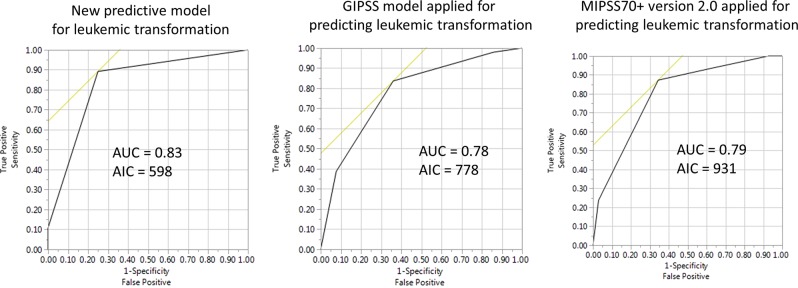
Predictive accuracy of the new risk model for leukemic transformation in primary myelofibrosis, compared to previously established overall survival risk models, including the genetically-inspired prognostic scoring system (GIPSS) and karyotype- and mutation-enhanced prognostic scoring system (MIPSS70 + version 2.0)

Using the results from the Cox time-to-event multivariable analysis, a predictive model for LT was devised, with point allocations commensurate with HR values; *IDH1* (HR 4.3; 3 points), circulating blasts ≥ 3% (HR 3.3; 2 points), *SRSF2* mutations (HR 3.0; 2 points), age > 70 years (HR 2.1; 1 point), *ASXL1* mutations (HR 2.0; 1 point) and sex-adjusted moderate or severe anemia (HR 1.9; 1 point). A total of 456 patients were informative for all six independent predictors of LT; subsequently a three-tiered LT risk stratification was developed: high-risk (7–8 adverse points; LT incidence 57%; HR 39.3, 95% CI 10.8–114), intermediate-risk (2–6 adverse points; LT incidence 17%; HR 4.1, 95% CI 2.4–7.3) and low-risk (0–1 adverse points; LT incidence 8%) (Fig. 1). AIC and AUC analysis confirmed the superior performance of the new LT predictive model (AIC 598; AUC 0.83), compared to GIPSS (AIC 778; AUC 0.78) and MIPSS70 + version 2.0 (AIC 931; AUC 0.79) for predicting LFS (Fig. 2).

## Discussion

Leukemic transformation (LT) is a dreaded complication of myeloproliferative neoplasms (MPN); reported 10-year estimates of LT incidence range from 0.7–3% for ET, 2.3–14.4% for PV and 10-20 % for PMF^2,16–18^. In a recent communication of 410 patients with post-MPN LT, recruited from the Mayo Clinic (*n* = 248) and multiple centers in Italy (*n* = 162), median survival was only 3.6 months and post-LT survival was independently affected by unfavorable karyotype, platelet count < 100 × 10^9^/l, age > 65 years and transfusion need at time of LT^19^. In general, long-term survival after LT was unusual, despite the achievement of close to 60% rate of complete remission, with or without incomplete count recovery^19^. The particular study revealed treatment-specified 3-year/5-year survival rates of 32%/10% for patients receiving allo-SCT, 19%/13% for patients achieving remissions following intensive chemotherapy but were not subsequently transplanted, and 1%/1% in the absence of both allo-SCT and chemotherapy-induced remission^19^. In other words, the survival benefit of allo-SCT in MPN^20^ might not extend to those with LT, which underscores the need to identify patients at risk, before they undergo LT.

Current prognostic models in PMF target OS and utilize both genetic and clinical risk factors: MIPSS70 (mutation-enhanced international prognostic scoring system for transplant-age patients)^7^, MIPSS70 + version 2.0 (karyotype-enhanced MIPSS70)^4^ and GIPSS (genetically-inspired prognostic scoring system)^3^. Both GIPSS and MIPSS70 + version 2.0 also predict LFS and the relevant risk factors in this regard included unfavorable karyotype, *SRSF2* and *ASXL1* mutations, platelet count < 100 × 10^9^/l and circulating blasts ≥ 2%^3,7^. Other previously cited risk factors for LT in PMF include age > 65 years^21^, red blood cell transfusion need^22^, leukocyte count > 30 × 10^9^/l^23^, platelet count < 50 × 10^9^/l^24^, circulating blasts ≥ 3%^25^, increased levels of serum IL-8 and IL-2R^26^, C-reactive protein > 7 mg/l^21^ and bone marrow blasts ≥ 10%^24^. LT in PMF has also been associated with certain chromosomal abnormalities, including chromosome 17 aberrations^24^, monosomal karyotype^27^ and unfavorable karyotype including complex karyotype and those affecting chromosomes 5 or 7^28–30^. More recent information suggests that patients with triple-negative driver mutational status^2^ and those who harbor *ASXL1*, *SRSF2*, *IDH1* or *IDH2* mutations^14^ were also at increased risk of LT.

The current study provides a highly discriminating LT predictive model for PMF, which was shown to be superior to both GIPSS and MIPSS70 + version 2.0 in its LT predictive accuracy (Fig. 2). However, it should be noted that almost all of the variables used in the new LT predictive model (i.e., *IDH1*, *ASXL1*, *SRSF2* mutations, circulating blasts ≥ 3%, age > 70 years and moderate/severe anemia) were previously associated with shortened LFS (*see above*). What is different in the current study was i) the much larger sample size of informative cases; ii) the distinction between early events (logistic analysis of LT risk in the first 5 years of diagnosis) and overall risk (assessed by Cox analysis of LFS); iii) the combined analysis of mutations, cytogenetic abnormalities and clinical variables, in order to decipher inter-independent risk factors; and iv) development of a novel LT predictive model that includes both genetic and clinical risk factors. From a practical standpoint, the new LT risk model for PMF complements GIPSS and MIPSS70 + version 2.0 and should provide additional layer of prognostic information to assist with treatment decision-making, especially in terms of patient selection for allo-SCT. The current study also confirms the prognostic importance of specific mutations, sex-adjusted anemia and excess circulating blasts, for both OS and LFS, in PMF. Our observations require further validation, which might not be easy to accomplish, considering the difficulty in securing adequate number of informative cases.
